# Compassion Fatigue, Satisfaction and Automated Medication Dispenser: A Pilot Mixed-Methods Study

**DOI:** 10.1093/geroni/igaf122.2647

**Published:** 2025-12-31

**Authors:** Karyman Ghanem, Bincy Baby, Colleen McMillan, Kristen Antunes, Kelly Grindrod, Annette McKinnon, Linda Lee, Tejal Patel

**Affiliations:** University of Waterloo, Waterloo, Ontario, Canada; University of Waterloo, Waterloo, Ontario, Canada; University of Waterloo, Waterloo, Ontario, Canada; Custom Health, Kelowna, British Columbia, Canada; University of Waterloo, Waterloo, Ontario, Canada; Patient Advisors Network, Toronto, Ontario, Canada; McMaster University, Hamilton, Ontario, Canada; University of Waterloo, Waterloo, Ontario, Canada

## Abstract

Informal caregivers frequently support older adults with complex medication management tasks. However, most informal caregivers are inadequately trained to manage these challenging tasks and may experience caregiver burden. The study aims to evaluate the impact of an automated medication dispenser (AMD) on compassion fatigue, satisfaction, and medication administration hassles. This study recruited 7 pairs of family caregivers and their older care recipients. Caregivers completed the Family Caregiver Medication Administration Hassles Scale (FCMAHS) and the Professional Quality of Life Scale (ProQoL) at baseline, 2 weeks, and 3 months after implementing AMD in care recipients’ homes. Friedman tests showed no significant change in FCMAHS subscale scores over time after Bonferroni correction (α = 0.0125; all p > 0.0125). The total score (primary outcome) was assessed without correction (α = 0.05) and was not significant. Wilcoxon Signed-Rank Tests showed a similar pattern, except for a significant reduction in total score from baseline to 3 months (p = 0.02). Both tests showed no significant change in scores for the subscales of ProQoL after Bonferroni correction (α = 0.0167); all p > 0.0167). Caregivers were interviewed before and after using AMD. The post-intervention interviews were recorded, transcribed, and thematically analyzed. Four themes emerged from the analysis: usability and functionality, experience with remotely delivered pharmacy services, caregiver experience and wellbeing, and impact on caregivers/recipient relationships. The long-term use of AMD has the potential to be beneficial for caregiving burden related to medication management but is influenced by the caregiver’s adjustment period. Future research should verify these pilot findings.

